# The Use of MoStBioDat for Rapid Screening of Molecular Diversity

**DOI:** 10.3390/molecules14093436

**Published:** 2009-09-08

**Authors:** Andrzej Bak, Jaroslaw Polanski, Agata Kurczyk

**Affiliations:** Institute of Chemistry, University of Silesia, Szkolna 9, 40007 Katowice, Poland; E-mail: polanski@us.edu.pl (J.P.)

**Keywords:** combinatorial chemistry, virtual screening, relational database, ligand, macromolecule, MoStBioDat

## Abstract

MoStBioDat is a uniform data storage and extraction system with an extensive array of tools for structural similarity measures and pattern matching which is essential to facilitate the drug discovery process. Structure-based database screening has recently become a common and efficient technique in early stages of the drug development, shifting the emphasis from rational drug design into the probability domain of more or less random discovery. The virtual ligand screening (VLS), an approach based on high-throughput flexible docking, samples a virtually infinite molecular diversity of chemical libraries increasing the concentration of molecules with high binding affinity. The rapid process of subsequent examination of a large number of molecules in order to optimize the molecular diversity is an attractive alternative to the traditional methods of lead discovery. This paper presents the application of the MoStBioDat package not only as a data management platform but mainly in substructure searching. In particular, examples of the applications of MoStBioDat are discussed and analyzed.

## 1. Introduction

The formation of the receptor-ligand system is a complex phenomenon whose exploration is a challenging aim of contemporary chemistry and pharmacology. The awareness of the receptor structure targeted by a complementary bioeffector is a crucial point for identifying the lead structures both by *in vitro* (combinatorial chemistry) and *in silico* (HTS) screening. In fact, the receptor structure data are becoming more and more often available, which makes structure based methods, e.g. receptor based RD-QSAR, increasingly popular [1]. Similarly, we can observe an enormous interest in probing drug-receptor interactions using a large number of ligands in conjunction with molecular docking. Virtual screening with fragment based docking is a recent example [2,3,4].

Such computer-assisted simulations are the most progressive topic in present day drug design. This demands however the processing of enormous amounts of data. In turn it is the proper aggregation and organization of a chemical information dataset that enables massive virtual screening (VS). This is however hampered by the lack of the unified data standards. Among the steepest barriers to overcome in high-throughput screening studies is the limited number of suitable repositories of stored drug and drug target data. By offering a uniform data storage and retrieval mechanism different data might be compared and exchanged easily. *ZINC* is an example of a “drug-like” SDF ensemble of ligand structures that can be directly used as a basic source of data for *Ligand* [5]. The current version of the *Ligand* database contains approximately seven million compounds [6]. *Chem DB* is another database that includes approximately 4.1 million commercially available compounds [7,8].

Generally, several database systems with macromolecular data are also available publicly, but only a few combine the receptor-ligand data together. Moreover, practically in all cases the access to these data is limited to the respective web sites. The main goal of the current investigations was to provide an integrated software system, namely, the MoStBioDat [9,10] platform, for storing data in a unified format with an ensemble of tools for data manipulation. The major advantage of this platform relies on the possibility of being installed locally with a pretty simple database driven by the Python [11] package installation procedure. Users can establish their own hardware/software environment and data to import with a set of tools for storage, access and exchange of biological data. The modular architecture of the Python package also enables the extension of the system with necessary functionalities in the future. The whole MoStBioDat software can be freely downloaded from our web site. In the present publication we are reporting the current state of the project development. Moreover, we discuss here the product efficiency when applied to the analysis of the molecular diversity and molecular similarity of small molecules. 

## 2. Methods

### 2.1. MoStBioDat architecture

**Figure 1:**
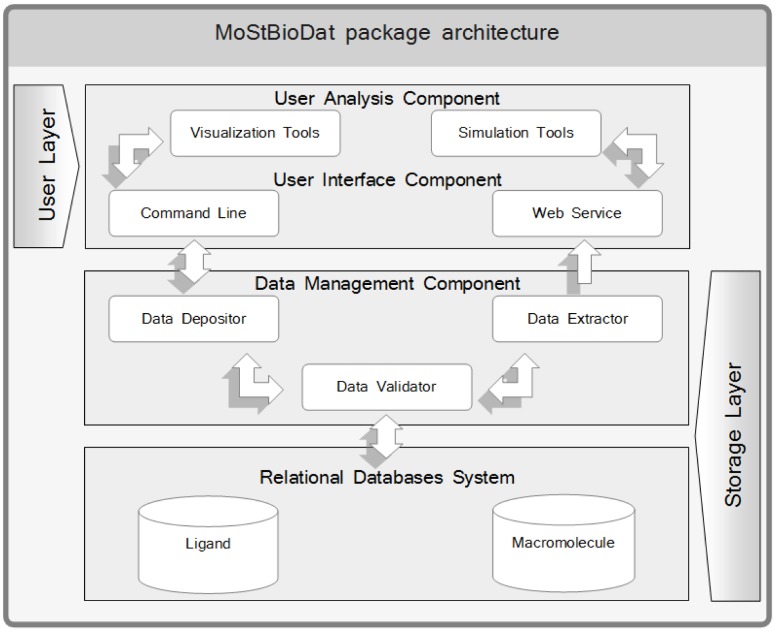
The architecture of the MoStBioDat package.

**Figure 2:**
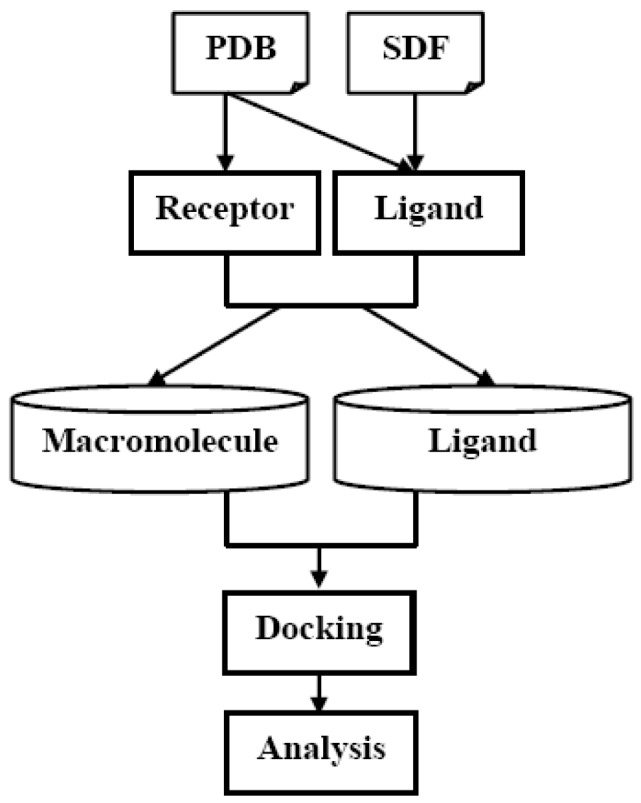
The basic virtual ligand screening pipeline.

A detailed description of the MoStBioDat system can be found elsewhere [9]. A brief overview is given in Figure 1. Conceptually, the system architecture encompasses Storage and User Layers, respectively. This system resembles the architecture of the BioSimGrid project, which has a similar structural layout [12,13]. Following the contemporary trends in the database technology the pragmatic relational approach has been applied to programming the Storage Layer that is in charge of storing and preserving data. Therefore, the database system has been designed to organize data relationally with parent-child key relationships that enable an efficient management of the stored datasets with the open-source MySQL as a database server [14]. The relational database system is composed of two major ingredients: *Ligand* and *Macromolecule*, respectively. The *Ligand* part consists of an ensemble of primary tables storing multiple molecule representations including line notation with the absolute SMILES code, protonation state and conformational sampling, respectively. The topology of the *Macromolecule* database reflects the conventional PDB flat-file structure and data hierarchy. It contains a set of tables to store and retrieve original PDB data. The entire system, including *Ligand* and *Macromolecule*, is integrated together combining the knowledge of small molecules and their corresponding drug targets (Figure 2). The underlying complexity of parsing, validating, storing and extracting data is hidden in the middleware called Data Management Component, which provides an abstract layer with sets of services responsible for making data transparently available. The post-processing component in the User Layer called User Interface Component offers two modes of browsing the databases. The users have access to datasets with fully programmable Python command line helpful for more specific analysis, whereas novice users can apply the graphical user interface (GUI) – not implemented yet in the current prototype. A set of pretty mature Python libraries and programs, for instance MMTK, VMD or R, are freely available with all necessary simulation and visualization tools [15,16,17]. Practically, the entire system is based on the client-server architecture, although the current prototype was implemented and tested where both applications and a database server was running at a single PC location with Intel Pentium 4 CPU 3.4 GHz, 2GB RAM memory and 1.5TB hard disk space available with GNU/Linux Debian 4.0 as a operating system. It should be emphasized that in the relationally designed systems the time/resource performance strongly depends on the manner of the query creation and the size of the database.

### 2.2. ZINC database

The ZINC database [5,6] was chosen as a basic set of structural data in order to avoid common problems with representation of compounds in the correct protonation, tautomeric and 3D conformational states. Moreover, ZINC offers the subsets of data especially prepared for docking. Thus, the data seem to be suitable to be integrated with macromolecular structures. 

### 2.3. Rapid screening of the ZINC database subset

The rapid examination of a large number of molecular data stored in a database combined with the calculation of the metric quantifying the similarity with a given pattern is one of the fundamental tasks in chemoinformatics. This is performed by the analysis of the entire structure or substructure, which form a search query for identifying the ensemble of compounds fulfilling the global similarity criteria.

In practice, similarity or diversity measures derived by comparing the presence and/or absence of features or the occurrences of substructures present in a molecule, are based on a abstract representations of certain structural features of a compound in form of the fixed-size binary fingerprint vector [18]. Characterizing a chemical structure in the binary form integrated with the efficient bit-wise algorithms yield high-speed structural screening procedure, which in comparison with the precise, computationally demanding exact search results might produce a bit higher rate of false positives [19]. 

Among different similarity measures and pattern matching procedures developed for molecular fingerprints applied as filtering methods identifying or eliminating drug-likeness of molecules, the Tanimoto coefficient [20,21] is the most common one. It defines the proportion of substructures in common between two molecules which is expressed by the following formula:

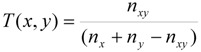
(1)
where:

n_xy_ - the number of bits set into 1 shared in the fingerprint of molecule x and y
n_x_ - the number of bits set into 1 in the molecule x
n_y_ - the number of bits set into 1 in the molecule y

Conceptually, the procedure of finding a particular pattern in a molecule might be interpreted as string regular expressions describing the search criteria. All compounds which share a common substructure might be identified using the straightforward extension of SMILES notation – the SMARTS pattern [20].

It is assumed that the largest common component that appears in structurally related drugs might determinate their biological activity. The Maximum Common Substructure (MCS) approach is an alternative method of pattern matching which provides a similarity score for a pair of structures used as a metric for ranking the molecular similarity [22,23]. The conversion of the MCS-based procedure into the CPU intensive maximum clique detection problem makes it impossible to be applicable in the high-speed database screening.

The analysis of some molecular properties allowed identification of those that are important for a drug’s pharmacokinetics (ADMET). The quantitative filter used for the drug-like molecule searches has also been formed by Lipinski’s Rule of Five (RO5) [24].The property space was restricted to the range of values defined by the octanol/water partition coefficient (ClogP ≤ 5), the molecular weight (MW ≤ 500), the number of hydrogen bond donors (HBD ≤ 5) and hydrogen bond acceptors (HBA ≤ 10), respectively. Roughly speaking, the violation of the above conditions might discriminate between prospective drugs and non-drugs, but RO5 does not represent precise rules sufficient for defining druglikeness [25]. Moreover, the distribution of the molecular weights for drugs and non drugs has also been neglected recently [26]. Taking into account more restrictive conditions (MW ≤ 460, -4 ≤ ClogP ≤ 4.2, HBD ≤ 5, HBA ≤ 9) the leadlikeness criteria has been established to identify drug prototypes, which are optimized before obtaining the drug candidate status [27]. The leadlike-based strategy is also expected to be applicable for database sampling as a integral part of the continual enrichment of the HTS procedure. 

## 2. Results and Discussion

**Figure 3:**
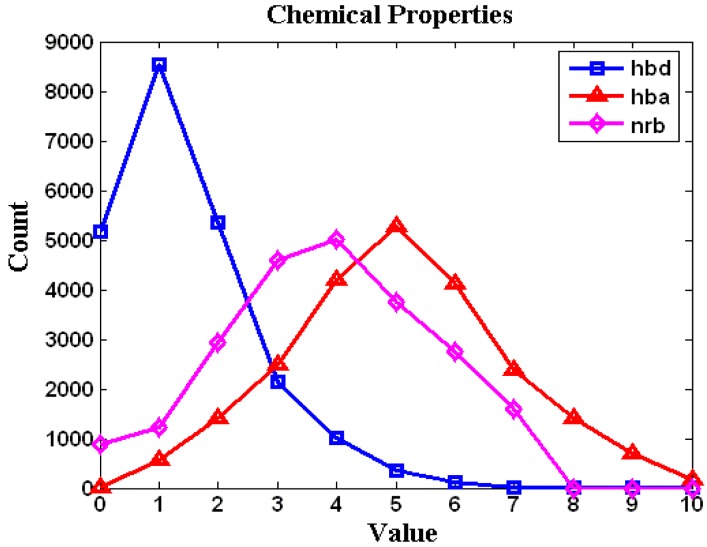
Histogram of some molecular descriptors calculated during screening of the *Ligand* database (hbd – H-bond donors, hba – H-bond acceptors, nrb – number of rotatable bonds).

**Figure 4:**
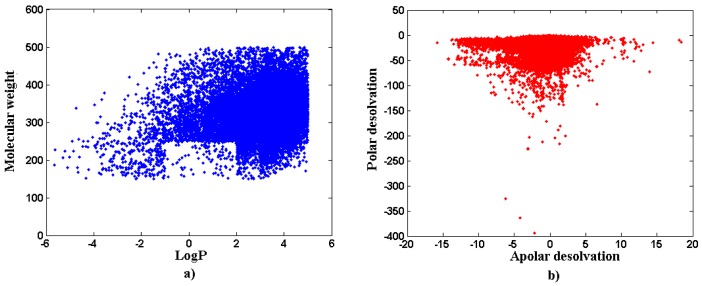
The distribution of the calculated logP *vs.* molecular weight (a) and apolar desolvation *vs.* polar desolvation (b).

The calculations of several pretty helpful statistics on small molecules (*Ligand* database), primarily intended as coding examples, have been conducted on the trial “drug-like” subset of 24,000 molecules taken from the ZINC database. The histogram counting the number of molecules in the function of commonly derived molecular descriptors such H-bond donors (hbd), H-bond acceptors (hba) or number of rotatable bonds (nrb) is shown in Figure 3. Additionally, the pairwise comparison displaying the mutual relationship between the calculated logP versus molecular weight and apolar desolvation versus polar desolvation are specified in Figure 4a and Figure 4b, respectively.

**Figure 5:**
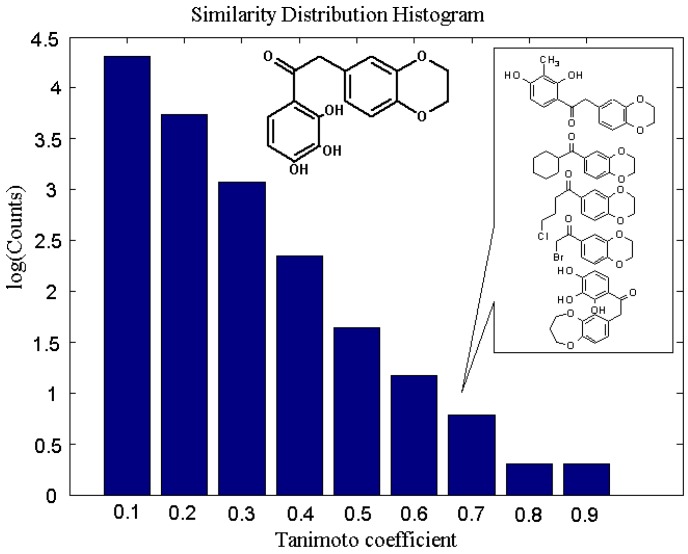
The histogram representing the similarity score distribution for the entire subset of molecules in the Ligand database calculated for the chosen search structure. The substructure bias, assessing the level of molecular similarity, is accomplished by setting the similarity measure ranging from 0.1 to 1.0. The examples of molecules with Tanimoto coefficient ≥ 0.7 are given in the internal frame.

The distribution of the structural similarity within the ensemble of small molecules to an arbitrary chosen molecule of 2-(2,3-dihydro-1,4-benzodioxin-6-yl)-1-(2,3,4-trihydroxyphenyl)ethanone indi-cated by its Tanimoto coefficient with respect to Daylight-type fingerprint is introduced in Figure 5. The substructure bias, assessing the level of molecular similarity, is accomplished by setting the similarity measure ranging from 0.1 to 1.0.

## 3. Experimental Section

### 3.1. Distribution

A brief description of the current development status along with the latest released version of the package to download and the generated documentation are available from the following web address*:* http://www.chemoinformatyka.us.edu.pl/mostbiodat/. Under this address the whole program can be downloaded for local installation on a user’s machine under the terms of a GPL version 3 license. The system can be installed on under the Linux system. To obtain full module functionality the academic OpenEye license is needed. The rest of the applied Python libraries are distributed under the GPL license.

### 3.2. Installation and operation of the MoStBioDat package

The system can be at the moment be downloaded from our website to be locally installed, operated and evaluated. This can be operated in a command line mode which is a limitation (mainly for beginners). It is very common to operate a variety of programs in such a manner. Being aware of the limitations of the command line access, especially for the beginners, future developments of the system will be equipped with a graphical user interface accessible via web browser. It should be emphasized that we have implemented the first prototype for evaluation and correction. Despite the lack of the GUI, it is still possible to establish a user-friendly text-mode environment using f.e. Eclipse with PyDev module. The MoStBioDat package with the ensemble of libraries may be regarded as a Python toolkit for data manipulation, what facilitates the program creation using some predefined blocks. This manner of programming is used in other packages f.e. mmLib, Pybel, rdKit, OEChem. The MoStBioDat package is still being developed and refined taking into consideration user remarks.

The listing below gives an example of the substructure search (cf. paragraph 2.3) using the Tanimoto coefficient and Lipinski’s Rule of Five:

A=DB2SmiDict(host=’localhost’,db=’ligand’,user=’’,passwd=’’,path=’/tmp/Log’,filename=’db2smi’)
  B=SubStructSearch(smidict=A.readb(logdebug=False),path=’/tmp/Log’,filename=’smisubsearch’)
Tanimoto Search ###
  B.TanimotoSearch(refsmile=’Cc1cccccc1’,coeff=0.7, outfile=’/tmp/TanimotoSearch.txt’)
Lipinski’s Rule of File Search ###
  B.RO5Search(outfile=’/tmp/RO5Search.txt’,MolWT=500,HBA=10,HBD=5,LogP=5)

### 3.3. Data format

There is no restriction imposed on the source of data – the only limitation is the format of the input files (sdf or pdb). One can decided to use other remote sites, e.g., PubChem or DrugBank as a supplementary source of data, generally providing physical, chemical or biological properties of compounds. The data providing the topographical description of the particular compound are generally distributed in the mol or sdf file format. Additional set of properties is frequently offered in a separate spreadsheet with the canonical SMILES code to identify the compound. We have implemented two modules for the spreadsheet parsing so far (MoStBioDat/DataBase/ImportData/Data2DB/**PropZINC**, MoStBioDat/DataBase/ImportData/Data2DB/**PropDrugBank**).

### 3.4. Substructure searches

The OpenBabel’s [28] and OpenEye’s [29] functions have been applied to generate and compare the molecular fingerprints and the SMARTS matching in the *SubstructureSearch* module (TanimotoSearch, SMARTSearch, QuerySearch, MCSearch, CliqueSearch, RO5Search).

## 4. Conclusions

The main goal of this project was to provide an integrated software system for storing data in a unified format with an ensemble of tools for data manipulation. Conceptually, we have followed some known solutions trying to gather them together. However, the MoStBioDat one is designed to include both the ligand and macromolecule data. Generally, several database systems are publicly available with macromolecular or ligand data, but only a very few combine them together. Moreover, for almost all of them, access to this data is limited to the corresponding web sites. The major advantage of the MoStBioDat platform relies on the possibility of being installed locally, which allows users to establish their own hardware/software environments and data to import with a set of tools for storage, access and exchange of the biological data. 

In the example of application we have shown the effective data manipulation within the “drug-like” subset of 24,000 molecules taken from ZINC database. This allowed for the rapid screening of the H-bond donors, H-bond acceptors, number of rotatable bonds. The logP *vs.* molecular weight and apolar desolvation *vs.* polar desolvation relationships can also be plotted and analyzed easily. Finally, the MoStBioDat platform makes available rapid screening in a search for related molecules on the basis of similarity scores. 
